# Longitudinal tumor response dynamics after staged gamma knife radiosurgery combined with bevacizumab in sporadic vestibular schwannoma

**DOI:** 10.1016/j.ctro.2026.101318

**Published:** 2026-10-14

**Authors:** Zeyu Peng, Shangbo Wang, Jiao-jiao Cao, Hongchi Zhang, Jin Yang, Liang-Yun Hu, Ran-ran Dai, Si-Jian Pan, Cheng-Jie Qiu

**Affiliations:** a Department of Neurosurgery, Ruijin Hospital, Shanghai Jiao Tong University School of Medicine, Shanghai, China; b Department of Pulmonary and Critical Care Medicine, Ruijin Hospital, Shanghai Jiao Tong University, School of Medicine, Shanghai, China

**Keywords:** Vestibular schwannoma, Gamma Knife radiosurgery, Bevacizumab, Tumor control, Pseudoprogression

## Abstract

**Background:**

Vestibular schwannomas (VSs) are benign tumors of the eighth cranial nerve. Although Gamma Knife radiosurgery (GKRS) achieves durable tumor control, delayed regression and pseudoprogression remain clinical challenges. Staged GKRS combined with bevacizumab may alter post-radiosurgical tumor response patterns.

**Aim:**

To compare longitudinal tumor response, pseudoprogression, and clinical outcomes between staged GKRS combined with bevacizumab and single-session GKRS alone in sporadic VSs.

**Methods:**

We retrospectively reviewed 72 patients with sporadic VSs: 49 treated with staged GKRS plus bevacizumab and 23 with single-session GKRS alone. Longitudinal tumor volume changes were analyzed using multivariable linear mixed-effects models, while early volumetric enlargement was evaluated using Kaplan-Meier estimates and Cox proportional hazards models.

**Results:**

Compared with GKRS alone, staged GKRS plus bevacizumab showed significantly different response trajectories (treatment-by-time interaction, *P* < 0.001), with earlier and sustained regression. Greater tumor reduction was observed from 3 to 36 months (all adjusted *P* < 0.05). Early volumetric enlargement (>10%) occurred in 14.3% versus 60.9% of patients (HR, 0.16; 95% CI, 0.06–0.40; *P* < 0.001). Pseudoprogression occurred in 4.1% versus 17.4% of patients, without statistical significance (*P* = 0.078). Cystic tumor composition was independently associated with greater regression. BEV-related adverse events were mild and manageable.

**Conclusion:**

Staged GKRS combined with bevacizumab was associated with earlier, more sustained tumor regression and a lower risk of early post-treatment enlargement in sporadic VSs. Larger baseline tumor volume and cystic composition were associated with greater regression. Pseudoprogression was numerically less frequent, although the difference was not statistically significant.

**Trial registration:**

ChiCTR2600131856 (Chinese Clinical Trial Registry).

## Highlights

• Staged GKRS plus bevacizumab was associated with earlier, sustained regression.
• Combination therapy showed a trend toward less pseudoprogression (4.1% vs 17.4%).
• Cystic tumor composition was associated with greater tumor regression.
• Cranial nerve function was preserved; bevacizumab adverse events were manageable.

## 1 Introduction

Vestibular schwannomas (VSs) are benign tumors of the eighth cranial nerve, and current management options include observation, microsurgical resection, and stereotactic radiosurgery (SRS) [1], [2]. Gamma Knife radiosurgery (GKRS) provides durable tumor control rates of approximately 90%–96% with preservation of cranial nerve function [3], [4].

Despite its high efficacy, tumor regression after GKRS is often delayed, and pseudoprogression characterized by transient radiographic enlargement remains a major clinical challenge [5], particularly in large Koos IV tumors where volume expansion may worsen mass effect and complicate treatment assessment [6], [7]. Strategies enabling earlier regression are therefore clinically desirable.

Bevacizumab (BEV), a monoclonal antibody targeting vascular endothelial growth factor (VEGF), has shown tumor volume reduction and hearing improvement in neurofibromatosis type 2 (NF2)-associated VSs [8], [9], [10]. The presence of VEGF/VEGFR signaling in sporadic VSs suggests potential applicability beyond NF2 disease; however, its role in sporadic VSs remains unclear [11].

Conventional single-session GKRS is limited by radiation tolerance of adjacent neural structures [12], whereas staged GKRS allows fractionated delivery and treatment of larger tumors [13], [14]. Combining staged GKRS with BEV may therefore promote tumor regression while preserving neurological function.

In this retrospective study, we compared longitudinal tumor response, pseudoprogression, neurological outcomes, and treatment-related adverse events between staged GKRS plus BEV and single-session GKRS in sporadic VSs. Particular attention was given to large and cystic tumors, in which early regression may be clinically beneficial.

## 2 Materials and methods

### 2.1 Participants and treatment

This single-center retrospective analysis was approved by Ruijin Hospital Ethics Committee, Shanghai Jiao Tong University School of Medicine, with a waiver of informed consent (approval number: (2025) Linlunshen No. (48)). The study was registered with the Chinese Clinical Trial Registry (ChiCTR2600131856).

Seventy-two patients with sporadic VSs diagnosed by magnetic resonance imaging (MRI) were included. Eligible patients underwent staged GKRS plus bevacizumab or single-session GKRS and had at least 6 months of follow-up. Patients with NF-2, bilateral VSs, age < 18 years, or incomplete clinical records were excluded.

Patients were categorized according to the treatment strategy received at our institution. Single-session GKRS was performed from January 2014 to June 2020, whereas staged GKRS plus bevacizumab was performed from August 2021 to November 2025. All radiosurgical procedures were performed by the same team using the Leksell Gamma Knife Perfexion or Icon system with Leksell GammaPlan software. Perfexion and Icon systems were used in 60 and 12 patients, respectively.

Within each period, treatment selection took into account tumor size, prior treatment, and patient preference. For staged GKRS, the entire tumor volume was treated at each session rather than being divided into separate spatial volumes. A prescription dose of 6–9 Gy per session was delivered in 2–5 sessions at one-month intervals, with a median cumulative dose of 24 Gy (IQR, 21–24 Gy). Single-session GKRS was delivered with a prescription dose of 11–15 Gy (median, 13 Gy; IQR, 12.5–13.5 Gy). A 50% isodose line was used, and target delineation was based on contrast-enhanced T1-weighted MRI without margin expansion. Dose constraints were < 9 Gy for the cochlea and < 15 Gy for the brainstem.

BEV was administered intravenously every 4 weeks at a median dose of 5 mg/kg (range, 3–7 mg/kg), beginning on the first GKRS session. Patients received a median of 9 cycles (range, 2–19), with a median cumulative dose of 41 mg/kg (IQR, 22.2–57.9 mg/kg). Among 14 patients receiving BEV for <6 months, treatment was discontinued shortly after completion of staged GKRS or within 1–2 months after GKRS according to patient preference because of concerns regarding potential adverse effects. No discontinuation was attributable to BEV-related toxicity. Contrast-enhanced MRI was performed before treatment and during follow-up. Tumor volumes were independently contoured on contrast-enhanced T1-weighted MRI using ITK-SNAP (version 3.8) by two blinded neurosurgical reviewers [15]. Tumor volume change was calculated as the percentage change from baseline. Pseudoprogression was defined retrospectively as a transient increase in tumor volume of >10% relative to baseline, followed by subsequent regression below the baseline volume, with no recurrent increase above baseline on available follow-up MRI.

### 2.2 Clinical outcomes and adverse events

Neurological outcomes and cranial nerve-related symptoms were assessed using outpatient medical records. Hearing outcomes were based on documented patient-reported status because standardized audiometric testing was not routinely available. Symptom changes were categorized as improved, stable, worsened, or new-onset. Bevacizumab-related adverse events were extracted from clinical records [16].

### 2.3 Statistical analysis

Continuous variables are presented as mean ± SD or median (range), and categorical variables as number (%). Between-group comparisons were performed using the Student's *t*-test or Mann-Whitney *U* test for continuous variables and the χ^2^ test or Fisher's exact test for categorical variables. Longitudinal tumor volume changes were analyzed using multivariable linear mixed-effects models with treatment, time, and treatment-by-time interaction as fixed effects, adjusting for z-standardized baseline volume, Koos grade, previous surgery, age, and sex. Time-to-event analyses using Kaplan-Meier curves and Cox proportional hazards models were performed for early volumetric enlargement. As a sensitivity analysis, stabilized inverse probability of treatment weighting (IPTW) based on propensity scores was performed. Propensity scores were estimated using logistic regression with baseline tumor volume, Koos grade, previous surgery, age, and sex as covariates; covariate balance after weighting was assessed using absolute standardized mean differences (SMDs). A two-sided *P* < 0.05 was considered statistically significant.

## 3 Results

### 3.1 Participant characteristics

49 patients were treated with staged GKRS plus bevacizumab and 23 patients treated with single-session GKRS were included (Fig. 1). Baseline characteristics of the two groups are summarized in Table 1.

**Fig. 1:**
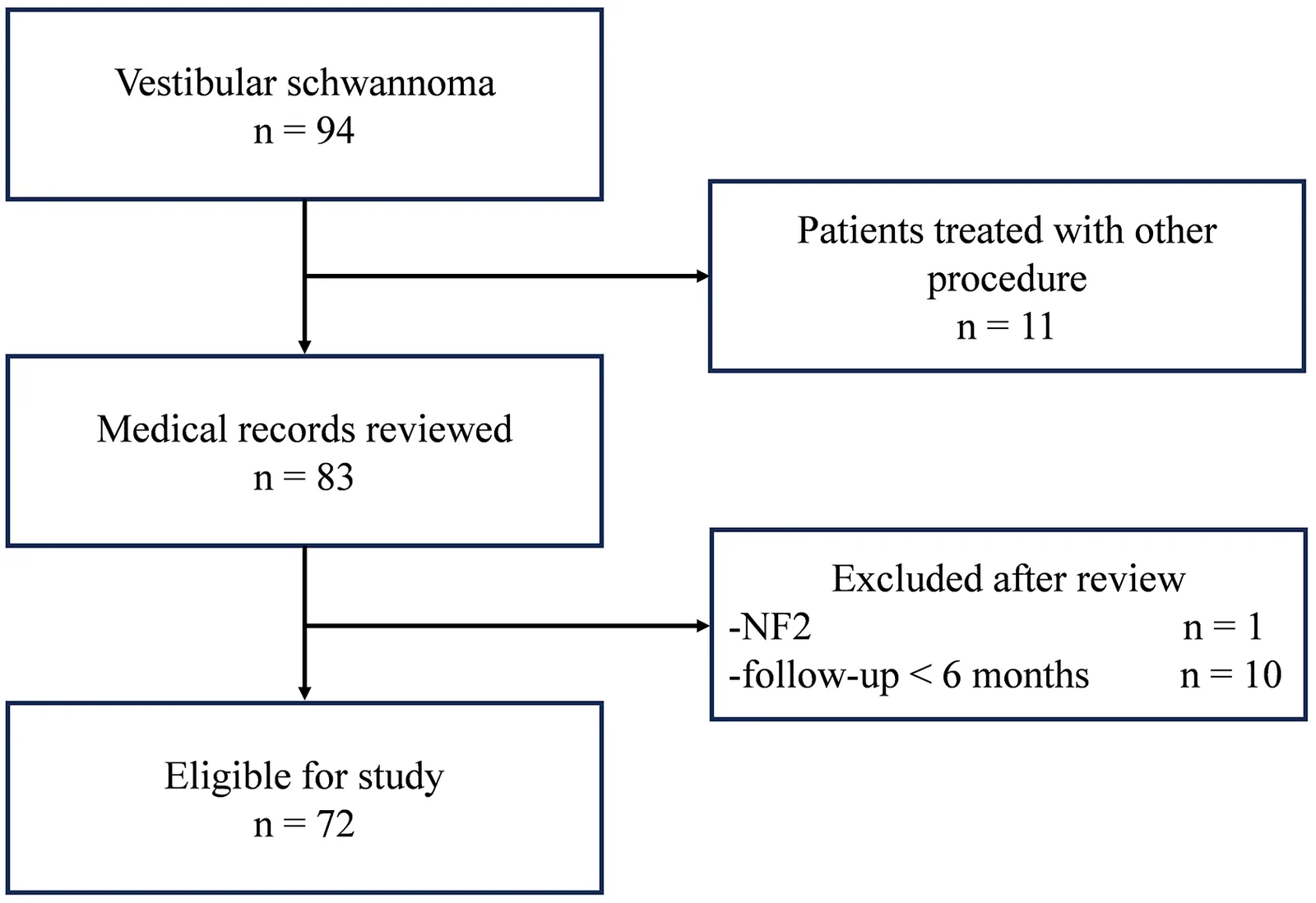
Flowchart of patient selection.

**Table 1:** Baseline Demographic and Clinical Characteristics of Patients Treated with staged GKRS Combined with Bevacizumab or GKRS Alone.

**Characters**	**Staged GKRS plus bevacizumab** **(*n* = 49)**	**GKRS alone** **(*n* = 23)**	***p*-value**
Age (yr)			0.990
median	54	50	
range	19–79	24–76	
Sex, No. (%)			1.000
Female	28 (57)	13 (57)	
Male	21 (43)	10 (43)	
Tumor volume			0.313
Mean (SD), mm^3^	4927 (4737)	6033 (4576)	
Median (range), mm^3^	3578 (103–23,090)	5436 (143–16,830)	
Koos grades, No. (%)			0.641
I	4 (8)	2 (9)	
II	9 (18)	3 (13)	
III	14 (29)	4 (17)	
IV	22 (45)	14 (61)	
Radiologic Classification (%)			0.799
Solid	27 (55)	14 (61)	
Cystic	22 (45)	9 (39)	
Initial symptoms			
Hearing decline	42 (86)	18 (78)	0.552
Tinnitus	21 (43)	8 (35)	0.590
Dizziness	22 (45)	14 (61)	0.207
Imbalance	9 (18)	7 (30)	0.340
Facial numbness	16 (33)	7 (30)	1.000
Facial palsy	11 (22)	5 (22)	1.000
Median prescription dose, Gy	8 (per session); 24 (cumulative)	13	
Median isodose line, %	50	50	
Median follow-up (range), m	18 (6–49)	18 (6–70)	
Indication for GKRS, n (%)			0.184
Primary treatment	29 (60)	18 (78)	
Adjuvant treatment	20 (40)	5 (22)	
Bevacizumab treatment			
Duration, Median (range)	9 (2–19)	0	
Median dose (range), mg/kg	5 (3–7)	0	
Cumulative dose(range), mg/kg	41 (7–97)	0	

Longitudinal tumor volume changes according to treatment strategy.

Tumor response trajectories differed between treatment groups (Fig. 2). In the multivariable-adjusted linear mixed-effects model, a significant treatment-by-time interaction was observed (F = 12.79, *P* < 0.001). The interaction remained significant in the propensity score-weighted sensitivity analysis (β = −0.010, *P* < 0.001).

**Fig. 2:**
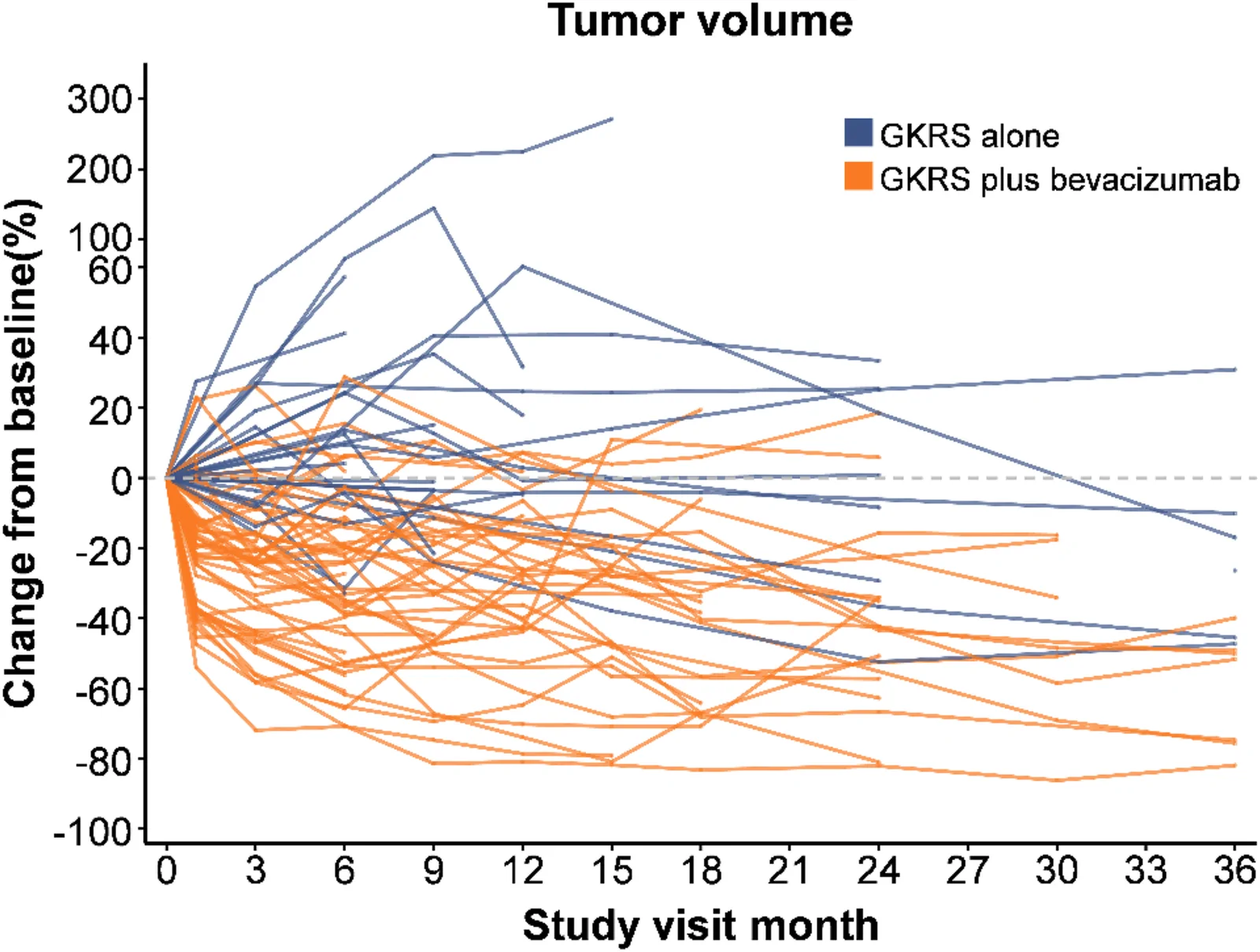
**Individual Tumor Volume Trajectories After staged GKRS Combined with Bevacizumab or GKRS Alone.** Tumor volume changes are presented as percentage changes from baseline. Patients were categorized according to treatment group.

Patients receiving staged GKRS plus bevacizumab demonstrated sustained tumor regression throughout follow-up. The observed mean tumor volume changes were − 19.6%, −27.6%, −28.7%, −34.9%, −32.2%, −38.1%, −42.3%, −40.0%, −47.6%, and − 60.4% at 1, 3, 6, 9, 12, 15, 18, 24, 30, and 36 months, respectively. Conversely, the GKRS alone group showed a fluctuating response pattern, with mean volume changes of +12.1%, +13.6%, +31.4%, +39.3%, +48.6%, and + 153.4% at 3, 6, 9, 12, 15, and 18 months, respectively, followed by regression to −2.6% and − 19.2% at 24 and 36 months. A representative case demonstrating continuous tumor regression after staged GKRS plus bevacizumab is shown in Fig. 3.

**Fig. 3:**
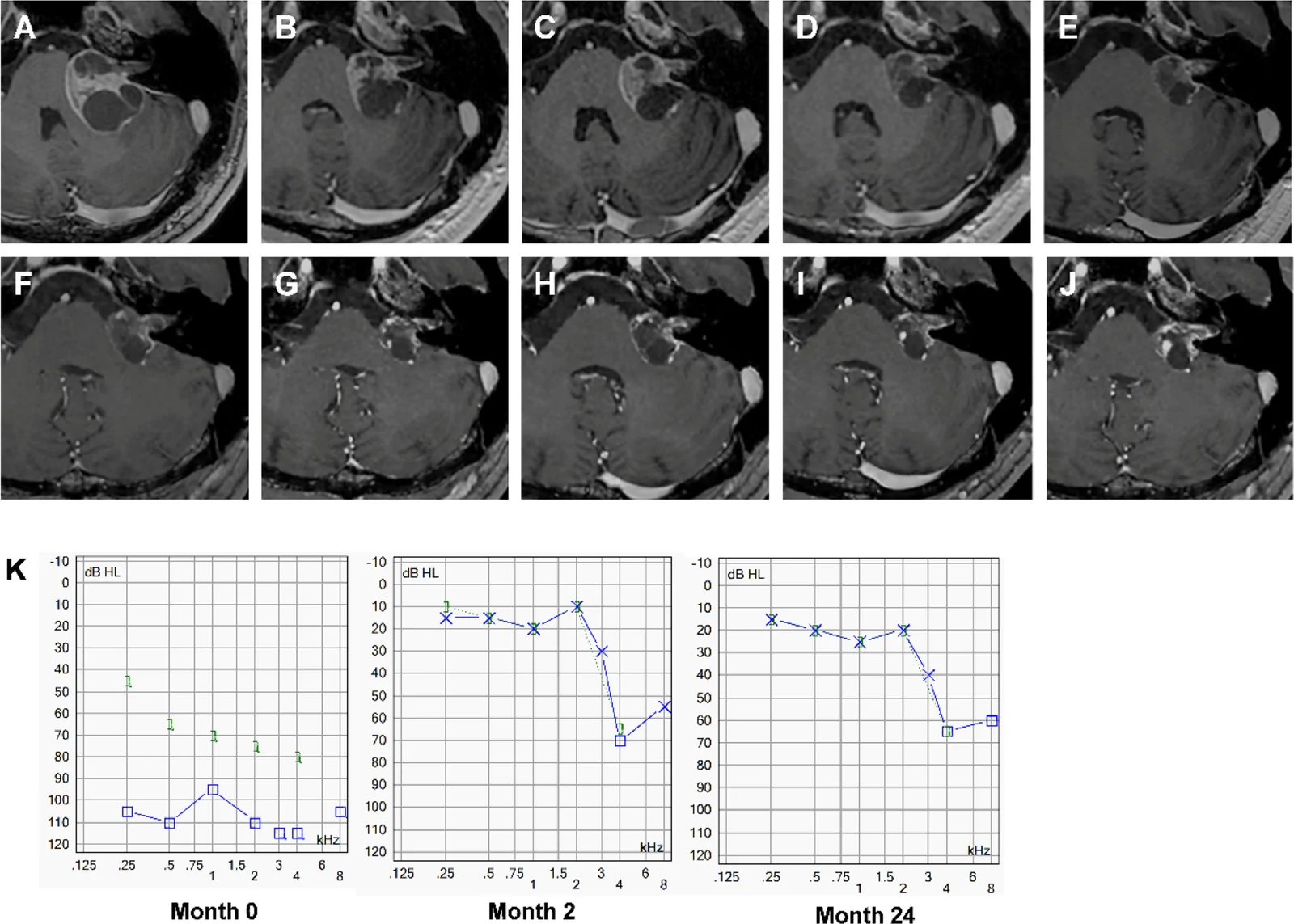
**Typical Magnetic Resonance Imaging (MRI) Response Following staged GKRS Combined with Bevacizumab.** Representative images from a 29-year-old male patient are shown. Panel A demonstrates the baseline tumor before treatment. Tumor volume reductions were calculated at 1, 4, 7, 10, 13, 16, 18, 21, and 24 months after treatment, corresponding to reductions of 43%, 56%, 71%, 81%, 81%, 82%, 80%, 83%, and 82%, respectively (Panels B–J). Panel K shows pure-tone audiometry (PTA) results before treatment and at 2 and 24 months after treatment.

Post hoc comparisons based on estimated marginal means demonstrated significantly greater tumor volume reduction in the staged GKRS plus BEV group at 3, 6, 9, 12, 15, 18, 24, and 36 months following FDR adjustment (all adjusted *P* < 0.05).

Patterns of pseudoprogression after treatment.

Interobserver variability was low, with a median absolute relative difference of 1.21% among 30 randomly sampled tumors, indicating good measurement consistency. Early volumetric enlargement (>10%) occurred in 14/23 (60.9%) GKRS patients versus 7/49 (14.3%) GKRS+BEV patients, with a significantly lower event risk in the GKRS+BEV group (HR, 0.16; 95% CI, 0.06–0.40; *P* < 0.001). After weighting, all baseline covariates were well balanced between groups (all absolute SMDs <0.10). After propensity score weighting, the association between treatment strategy and early volumetric enlargement remained significant (HR, 0.15; 95% CI, 0.06–0.40; *P* < 0.001). Similar associations were observed in sensitivity analyses using more conservative thresholds of >15% (HR, 0.19; 95% CI, 0.06–0.55; *P* = 0.002) and > 20% (HR, 0.13; 95% CI, 0.04–0.49; *P* = 0.002) for early volumetric enlargement. Among patients with enlargement, the median onset was 6 months in both groups (*P* = 0.878), while the median peak increase was 31.2% (IQR, 17.4%–53.3%) versus 19.4% (IQR, 14.7%–24.6%) (*P* = 0.080).

According to the predefined criteria, pseudoprogression occurred in 4/23 (17.4%) GKRS patients and 2/49 (4.1%) GKRS+BEV patients (*P* = 0.078). Median time to regression below baseline was 16.5 and 15 months, respectively, with no significant between-group difference.

Tumor characteristics associated with response after GKRS combined with bevacizumab.

Within the GKRS+BEV cohort, baseline tumor characteristics associated with longitudinal regression were assessed using linear mixed-effects models. Koos classification was associated with regression trajectories (Koos-by-time interaction: F = 2.66, *P* = 0.004), but not with overall volume change after adjustment for baseline volume and clinical covariates (F = 0.30, *P* = 0.589). Larger baseline tumor volume was associated with a more pronounced volumetric reduction trajectory over time (volume-by-time interaction: F = 4.56, *P* < 0.001). Cystic tumors showed greater regression than solid tumors (composition-by-time interaction: F = 6.33, *P* < 0.001), with significant differences from 3 to 30 months after treatment (all *P* < 0.05) (Fig. 4***)***.

**Fig. 4:**
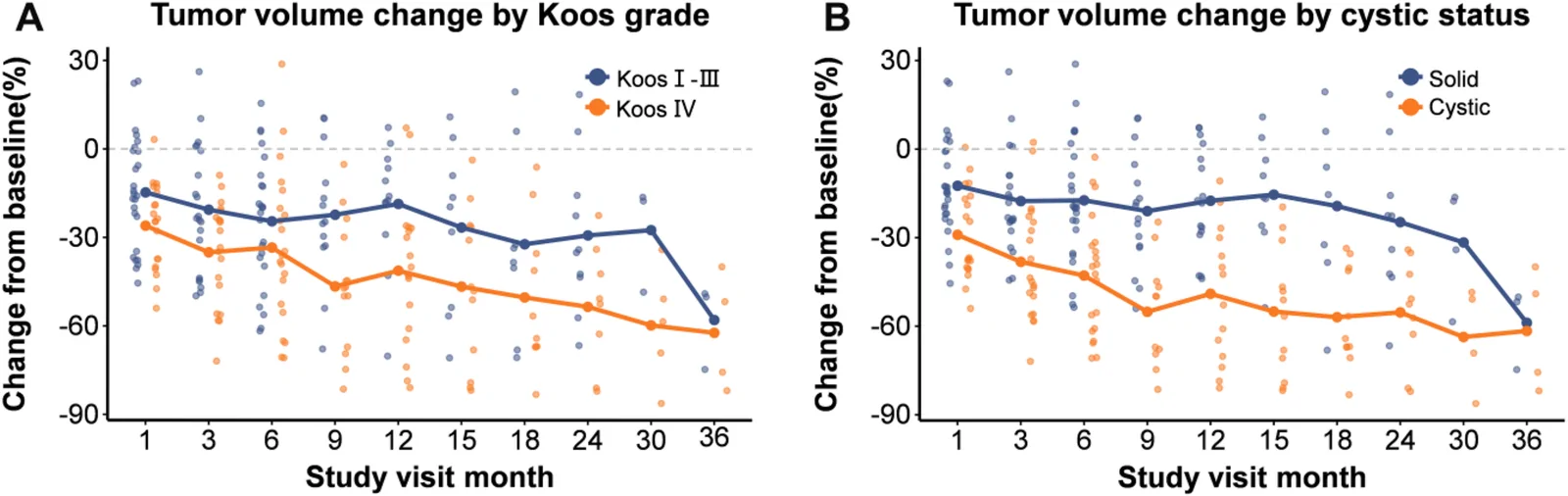
**Tumor Volume Reduction After staged GKRS Combined with Bevacizumab According to Koos Classification and Tumor Composition.** (A) Comparison of percentage volume reduction between Koos I–III and Koos IV VSs (unadjusted comparison). Koos IV tumors showed greater volume reduction at 3, 9, 12, 15, 24, and 30 months (all *P* < 0.05). (B) Comparison of percentage volume reduction between solid and cystic VSs. Cystic tumors showed greater volume reduction than solid tumors during the first 30 months of follow-up (all *P* < 0.05).

### 3.2 Clinical outcomes and neurological symptoms after treatment

Clinical outcomes are summarized in Table 2. Neurological function was generally maintained after staged GKRS combined with bevacizumab, with no significant deterioration observed during follow-up. Hearing status remained stable or improved in most patients, and new-onset cranial nerve-related symptoms were infrequent.

**Table 2:** Neurological Symptom Outcomes of Patients Treated with staged GKRS Combined with Bevacizumab or GKRS Alone.

**Clinical outcomes**	**Staged GKRS plus bevacizumab** **(n = 49)**	**GKRS alone** **(n = 23)**	***p*-value**
Hearing status, No. (%)			0.339
Absent throughout follow-up	6 (12)	2 (9)	
Improved	7 (14)	1 (4)	
Stable	29 (59)	14 (61)	
Worsened	6 (12)	3 (13)	
New-onset	1 (2)	3 (13)	
Tinnitus, No. (%)			0.479
Absent throughout follow-up	25 (51)	11 (48)	
Improved	7 (14)	1 (4)	
Stable	11 (22)	6 (26)	
Worsened	3 (6)	1 (4)	
New-onset	3 (6)	4 (17)	
Dizziness, No. (%)			0.183
Absent throughout follow-up	26 (53)	8 (35)	
Improved	9 (18)	3 (13)	
Stable	12 (25)	8 (35)	
Worsened	1 (2)	3 (13)	
New-onset	1 (2)	1 (4)	
Imbalance, No. (%)			0.595
Absent throughout follow-up	36 (73)	14 (61)	
Improved	3 (6)	1 (4)	
Stable	5 (10)	5 (22)	
Worsened	1 (2)	1 (4)	
New-onset	4 (8)	2 (9)	
Facial numbness, No. (%)			0.251
Absent throughout follow-up	32 (65)	15 (65)	
Improved	6 (12)	0 (0)	
Stable	9 (18)	5 (22)	
Worsened	1 (2)	2 (9)	
New-onset	1 (2)	1 (4)	
Facial palsy, No. (%)			0.636
Absent throughout follow-up	38 (78)	17 (74)	
Improved	2 (4)	0 (0)	
Stable	7 (14)	4 (17)	
Worsened	2 (4)	1 (4)	
New-onset	0 (0)	1 (4)	

### 3.3 Treatment-related adverse events

Bevacizumab-related adverse events (AEs) were recorded during follow-up and are summarized in Table 3. Nine types of adverse events were observed, with most events being mild and manageable. The most frequently reported adverse events were hypertension (9/49, 18%), headache (5/49, 10%), bleeding (5/49, 10%), fatigue (4/49, 8%), and irregular menstruation (2/28, 7%). Less frequently observed events included nausea (1/49, 2%), abdominal pain (1/49, 2%), arthralgia (2/49, 4%), and oral mucositis (2/49, 4%). No cases of proteinuria were observed during follow-up.

**Table 3:** Bevacizumab-Related Adverse Events in Patients with Sporadic Vestibular Schwannomas.

ADEs	No. (%)
Hypertension	9(18)
Headache	5(10)
Bleeding	5(10)
Fatigue	4(8)
Irregular menstruation⁎	2(7)
Arthralgia	2(4)
Oral mucositis	2(4)
Nausea	1(2)
Abdominal pain	1(2)
Proteinuria	0(0)

⁎ The percentage was calculated based on 28 female participants with normal menstruation at baseline. All events were grade 1–2 according to CTCAE v5.0.

## 4 Discussion

In this study, we investigated the impact of staged GKRS combined with bevacizumab on longitudinal tumor response in sporadic vestibular schwannomas. The major findings were: (1) staged GKRS combined with bevacizumab altered longitudinal tumor response trajectory compared with single-session GKRS alone; (2) staged GKRS combined with bevacizumab was associated with a significantly lower risk of early post-treatment tumor enlargement, with a numerically lower magnitude and frequency of pseudoprogression (*P* = 0.078); (3) tumor volume and composition were associated with heterogeneous regression patterns after combined therapy; and (4) neurological symptoms remained generally stable with acceptable toxicity.

Bevacizumab, an anti-angiogenic monoclonal antibody targeting VEGF, was initially approved by the FDA in 2004 for the treatment of several malignancies [17]. Subsequent clinical studies demonstrated that bevacizumab could reduce tumor burden and improve hearing outcomes in patients with NF2-associated VSs [8], [10], [18]. Because VEGF/VEGFR signaling is also present in sporadic VSs and has been associated with tumor growth rate [19], bevacizumab may represent a potential therapeutic strategy beyond NF2-associated disease.

In the present study, staged GKRS combined with bevacizumab was associated with distinct post-radiosurgical tumor response trajectories compared with GKRS alone. Unlike the fluctuating response pattern commonly observed after GKRS alone, patients receiving combination therapy demonstrated early and sustained tumor regression during follow-up. The significant treatment-by-time interaction in the multivariable-adjusted linear mixed-effects model indicates that the combination strategy was associated with differences in both the magnitude and trajectory of tumor volume changes after radiosurgery.

Pseudoprogression represents an important clinical challenge after radiosurgery, as transient tumor enlargement may mimic treatment failure and potentially exacerbate mass effect-related symptoms, particularly in larger tumors [20]. In our cohort, patients receiving staged GKRS plus bevacizumab demonstrated a significantly lower risk of early post-treatment volumetric enlargement (14.3% vs. 60.9%, HR, 0.16, 95% CI, 0.06–0.40, *P* < 0.001). This association remained significant after stabilized propensity score weighting, although residual confounding related to treatment era and other unmeasured factors cannot be completely excluded. Consistently, the proportion of patients subsequently exhibiting a pseudoprogressive trajectory was numerically lower in the combination group. However, the timing and pattern of regression after volumetric enlargement were comparable between the two groups, suggesting that staged GKRS plus bevacizumab is primarily associated with a lower occurrence of post-radiosurgical volume expansion rather than altering the subsequent natural course of pseudoprogression.

The differences in post-radiosurgical volumetric trajectories may partly reflect variations in tumor characteristics [21]. In our cohort, cystic tumors tended to show more pronounced regression after staged GKRS combined with bevacizumab, and larger baseline tumor volume was likewise associated with a greater degree of volume reduction. Although these findings require prospective validation, tumor morphology and baseline volume may provide preliminary clinical clues for identifying patients who are more likely to achieve a favorable volumetric response and may help inform patient selection for combined treatment.

Neurological function remained generally stable in both groups, with no significant between-group differences observed during follow-up. Further prospective studies incorporating standardized longitudinal functional assessments are warranted to more comprehensively evaluate functional outcomes after treatment.

The observed volumetric response may involve two complementary mechanisms. First, bevacizumab may directly inhibit tumor growth through suppression of VEGF signaling [22]. Second, it may modify the post-radiosurgical response by reducing radiation-induced vascular permeability and subsequent transient tumor enlargement. Vascular normalization and improvement of the tumor microenvironment may also enhance the antitumor effects of GKRS [23]. These mechanisms may partly explain the lower risk of early enlargement and the more consistent tumor regression observed in the combination group.

The use of bevacizumab in vestibular schwannomas has mainly been explored in NF2-associated disease, while evidence in sporadic tumors remains limited. Our findings suggest that staged GKRS combined with bevacizumab may offer a favorable volumetric response in sporadic VSs, particularly in tumors with larger baseline volume or cystic composition. Prospective multicenter studies are warranted to validate these findings and further define the role of this combined treatment strategy.

## 5 Conclusion

Staged GKRS combined with bevacizumab was associated with earlier and more sustained tumor regression compared with GKRS alone in patients with sporadic VSs. The combination strategy was associated with a significantly lower risk of early post-treatment tumor enlargement and showed numerically lower frequency and magnitude of pseudoprogression, although the difference in pseudoprogression did not reach statistical significance. Larger baseline tumor volume and cystic tumor composition were associated with greater tumor regression after combined therapy. Neurological outcomes were generally stable, and bevacizumab-related adverse events were predominantly mild and manageable.

### 5.1 Limitations

Several limitations should be acknowledged. First, the two groups differed in both radiosurgical strategy and bevacizumab exposure, precluding attribution of the observed differences to bevacizumab alone. The absence of a staged-GKRS-alone cohort and the treatment-era differences also leave potential residual confounding. Second, the single-center retrospective design and modest sample size limit generalizability. Third, neurological outcomes, particularly hearing, were largely based on patient-reported symptoms rather than standardized testing. Finally, the median follow-up of 18 months was relatively short for assessing long-term tumor control and late toxicity. Prospective multicenter studies with standardized functional assessments are needed.

## CRediT authorship contribution statement

**Zeyu Peng:** Writing – original draft, Validation, Formal analysis. **Shangbo Wang:** Writing – original draft, Validation, Formal analysis. **Jiao-jiao Cao:** Visualization, Investigation, Data curation. **Hongchi Zhang:** Visualization, Investigation, Data curation. **Jin Yang:** Visualization, Investigation, Data curation. **Liang-Yun Hu:** Visualization, Investigation, Data curation. **Ran-ran Dai:** Writing – review & editing, Supervision, Resources, Project administration, Methodology, Conceptualization. **Si-Jian Pan:** Writing – review & editing, Supervision, Resources, Project administration, Methodology, Conceptualization. **Cheng-Jie Qiu:** Writing – review & editing, Supervision, Resources, Project administration, Methodology, Conceptualization.

## Ethics declaration

### Informed consent and patient details

The authors have NOT obtained informed consent from participants or their legal representatives. This was a retrospective study based on existing medical records and imaging data. The requirement for informed consent was waived by the Ruijin Hospital Ethics Committee, Shanghai Jiao Tong University School of Medicine (approval number: (2025) Linlunshen No. (48)) because of the retrospective nature of the study. The privacy rights of the patients were strictly observed.

### Studies in human

This study was performed in compliance with relevant laws, regulatory frameworks and guidelines where the research took place. This study was approved by the Ruijin Hospital Ethics Committee, Shanghai Jiao Tong University School of Medicine. (Approval No. (2025) Linlunshen No. (48)).

## Funding sources

This research did not receive any specific grant from funding agencies in the public, commercial, or not-for-profit sectors.

## Declaration of competing interest

The authors have no relevant financial or non-financial interests to disclose.
